# Hepatitis and tuberculosis testing are much less common than HIV testing among adults in Kisumu, Kenya: results from a cross-sectional assessment

**DOI:** 10.1186/s12889-021-11164-2

**Published:** 2021-06-15

**Authors:** Joshua Tunnage, Adam Yates, Chiaka Nwoga, Valentine Sing’oei, John Owuoth, Christina S. Polyak, Trevor A. Crowell, for the RV393 Study Group, Rachel Adongo, Rachel Aguttu, Hosea Akala, Julie Ake, Michael Bondo, Erica Broach, Christine Busisa, Nate Copeland, Jessica Cowden, Mark de Souza, Leigh Anne Eller, Milicent Gogo, Zebiba Hassen, Dale Hu, Michelle Imbach, Anne Juma, Oscar Kasera, Qun Li, Margaret Mbuchi, Mark Milazzo, Kayvon Modjarrad, Eric Ngonda, Jacob Nyariro, Jew Ochola, Roseline Ohore, Thomas Okumu, Mary Omondi, Timothy Omondi, Linnah Ooro, Beatrice Orando, June Otieno, Victorine Owira, Roselyn Oyugi, Merlin Robb, Eric Rono, Chi Tran, Hannah Turley

**Affiliations:** 1 U.S. Military HIV Research Program, Walter Reed Army Institute of Research, Silver Spring, MD USA; 2 Henry M. Jackson Foundation for the Advancement of Military Medicine, Bethesda, MD USA; 3 HJF Medical Research International, Kisumu, Kenya; 4 Army Medical Research Directorate—Africa, Kisumu, Kenya

**Keywords:** HIV, Hepatitis, Tuberculosis, Testing practices, Screening practices, HIV testing, Africa, Voluntary counseling and testing, Early diagnosis, Healthcare acceptability

## Abstract

**Background:**

Kenya has a high burden of HIV, viral hepatitis, and tuberculosis. Screening is necessary for early diagnosis and treatment, which reduces morbidity and mortality across all three illnesses. We evaluated testing uptake for HIV, viral hepatitis, and tuberculosis in Kisumu, Kenya.

**Methods:**

Cross-sectional data from adults aged 18–35 years who enrolled in a prospective HIV incidence cohort study from February 2017 to May 2018 were analyzed. A questionnaire was administered to each participant at screening for study eligibility to collect behavioral characteristics and to assess prior testing practices. Among participants without a history of previously-diagnosed HIV, multivariable robust Poisson regression was used to estimate prevalence ratios (PRs) and 95% confidence intervals (CIs) for factors potentially associated with HIV testing in the 12 months prior to enrollment. A hierarchical model was used to test for differential access to testing due to spatial location.

**Results:**

Of 671 participants, 52 (7.7%) were living with HIV, 308 (45.9%) were female, and the median age was 24 (interquartile range 21–28) years. Among 651 (97.0%) who had ever been tested for HIV, 400 (61.2%) reported HIV testing in the past 6 months, 129 (19.7%) in the past 6–12 months, and 125 (19.1%) more than one year prior to enrollment. Any prior testing for viral hepatitis was reported by 8 (1.2%) participants and for tuberculosis by 51 (7.6%). In unadjusted models, HIV testing in the past year was more common among females (PR 1.08 [95% CI 1.01, 1.17]) and participants with secondary education or higher (PR 1.10 [95% CI 1.02, 1.19]). In the multivariable model, only secondary education or higher was associated with recent HIV testing (adjusted PR 1.10 [95% CI 1.02, 1.20]). Hierarchical models showed no geographic differences in HIV testing across Kisumu subcounties.

**Conclusions:**

Prior HIV testing was common among study participants and most had been tested within the past year but testing for tuberculosis and viral hepatitis was far less common. HIV testing gaps exist for males and those with lower levels of education. HIV testing infrastructure could be leveraged to increase access to testing for other endemic infectious diseases.

## Background

HIV, viral hepatitis, and tuberculosis are major drivers of morbidity and mortality worldwide, with a disproportionate burden of disease in resource-limited settings such as sub-Saharan Africa [1]. There are approximately 38 million people living with HIV (PLWH) and HIV accounted for approximately 690,000 deaths worldwide in 2019, including approximately 440,000 in Africa [1, 2]. Hepatitis B virus (HBV) is carried by 240 million people, including up to a quarter of residents of high endemic African countries such as Zimbabwe [3], and contributes to mortality via cirrhosis, liver failure and hepatocellular carcinoma with increasing HBV-related mortality in Africa over the last two decades [4]. Hepatitis C virus (HCV) contributes to 499,000 deaths worldwide annually [5], including over 100,000 deaths attributed to viral hepatitis-related cirrhosis in Africa [6]. One quarter of the global population is infected with tuberculosis, which caused approximately 1.4 million deaths in 2019, including about 208,000 in PLWH. Over 547,000 of all deaths occurred in the African Region [7]. Coinfection with HIV and other infectious diseases is common, complicates disease management, and worsens outcomes.

HIV/HBV and HIV/HCV coinfection are each associated with accelerated progression of liver fibrosis as compared to viral hepatitis alone and heightened risk of extrahepatic complications [8, 9]. In patients with tuberculosis, HIV is a strong predictor of mortality and undiagnosed tuberculosis is the most common HIV-related cause of death. Coinfection also places an increased burden on healthcare systems, leading to higher resource utilization even in resource-rich environments [10]. Screening for each of these diseases is therefore essential for vulnerable populations affected by overlapping epidemics and pandemics in order to reduce their morbidity, mortality, and economic burden.

Kenya represents the largest economy in eastern and central Africa but has a high burden of each of these diseases. Kenya has an HIV prevalence of approximately 4.9%, and in PLWH the seroprevalence of HCV is approximately 10% [11]. Overall HBV seroprevalence ranges from 5 to 30%, depending on the population studied, and HCV seroprevalence is around 2.8% [6, 12]. Kenya ranks among the 20 countries with the highest tuberculosis incidence in the world [7].

Timely screening for HIV, viral hepatitis, and tuberculosis improves mortality across all three illnesses. Delayed screening and recognition of HIV status results in higher mortality from HIV-related illnesses, lower CD4 counts at diagnosis, and higher inpatient mortality when admitted for HIV-related illnesses [13]. Routine HBV screening has been shown to be cost-effective and improves outcomes in other populations with similar HBV prevalence to Kenya [14]. There is a safe and efficacious vaccine that can prevent HBV if administered prior to exposure, but in high endemic countries, it is critical to receive the vaccination early, to include the HBV birth dose [15]. Kenyan guidelines have included childhood vaccination against HBV since 2002 and now include vaccination for unexposed adults as well, but implementation of these vaccination guidelines has been incomplete in many communities [16, 17]. Additionally, screening prior to vaccination is necessary in adults as mass vaccination campaigns without prior screening have not been shown to be cost-effective [14].

In Kenya, HBV testing is recommended upon entry into care for all PLWH and also for people with specific risk factors such as infants born to high risk mothers, health care workers, hemodialysis patients, injection drug users, and those with high risk sexual behavior [17, 18]. Similarly, Kenyan guidelines indicate that everyone should be screened at least once for HCV, but in settings where routine screening is not possible, HCV screening should be prioritized for blood donors, hemodialysis patients, health care workers, intravenous drug users, people with high risk behavior, immunocompromised patients, people with pre-existing liver disease, and PLWH [17, 18]. Intensified case finding for tuberculosis is recommended for all Kenyan PLWH at each routine HIV care visit, which begins with a symptom-based clinical screening procedure that, if positive, is followed by laboratory-based testing [18]. For those without HIV, tuberculosis screening also begins with symptom assessment followed by laboratory testing as clinically indicated [19].

In order to facilitate screening of all individuals at risk for HIV, viral hepatitis, and tuberculosis, knowledge of the social determinants of prior testing is necessary. Prior studies in sub-Saharan Africa have shown that HIV testing is more common among individuals with exposure to blood donation and testing campaigns, access to treatment facilities, HIV knowledge and awareness, and collective perceived benefits of testing and treatment. Factors that appear to hinder HIV testing include inaccurate risk perception, fear of testing positive, and fear of stigma and discrimination within the local community [20, 21]. Of note, much of the data on determinants of testing come from studies in either resource-rich environments or from South Africa, which may not be generalizable to resource-limited settings in other parts of sub-Saharan Africa.

We evaluated factors associated with prior testing for HIV, viral hepatitis, and tuberculosis among people with and without HIV in Western Kenya. Given the constraints of working in a resource-limited area, these data may help concentrate testing in areas or populations that have been overlooked in prior studies or programs. Given the common routes of transmission, high disease burden, and worsened clinical outcomes with multiple infections, the development of strategies to improve early diagnosis and treatment of these three diseases may improve morbidity and mortality in this region.

## Methods

### Study population

These analyses were conducted using data from the RV393 prospective observational cohort in the Kisumu region of Kenya, which was conducted to determine HIV incidence and to inform HIV vaccine development. Male and female participants were eligible for enrollment if they were at risk for HIV, were aged 18–35 years, and reported sex with at least two partners in the preceding three months. These criteria were developed based on prior studies from the region demonstrating high HIV incidence among young adults with multiple sexual partners [22–24]. A small group of PLWH who satisfied other inclusion criteria were enrolled as a comparator group and to mask the HIV status of participants due to substantial HIV-related stigma in the community. Participants were excluded from the study if they had previously received investigational agents that could change HIV risk such as monoclonal antibodies or candidate HIV vaccines. Study participants were recruited via community outreach and mobilization activities facilitated by community-based organizations, local ministry of health offices, and community leaders. Targeted recruitment was conducted at locations known to be focal points for activities associated with HIV risk including bars, markets, and fishing villages as well as healthcare settings such as HIV testing centers and family planning clinics.

All participants provided written or witnessed informed consent in English, Kiswahili or Luo prior to enrollment. The study protocol was approved by institutional review boards at the Walter Reed Army Institute of Research, Silver Spring, Maryland (WRAIR #2290) and the Kenya Medical Research Institute, Nairobi, Kenya (SSC 3307).

### Study procedures

Data for these cross-sectional analyses were collected over screening and enrollment visits approximately 28 days apart. A complete medical history was taken and physical examination was performed. Participants completed comprehensive questionnaires that included questions about sexual and other HIV risk behaviors as well as questions about prior testing. In three separate questions, participants were asked if they had ever been tested for HIV, hepatitis and tuberculosis. If the answer to a question was affirmative, participants were asked when the last test for that infection was performed with answer choices that included less than 6 months ago, between 6 and 12 months ago or more than 1 y ago. They were also asked if they had ever been treated or were currently being treated for HIV, viral hepatitis, and tuberculosis. A social history was taken that included documentation of any history of alcohol abuse, defined as having more than 5 alcoholic drinks in 1 d and/or causing harm while intoxicated. Participants were screened for symptoms of possible tuberculosis including cough, fever, weight loss, chest pain or breathlessness, or night sweats of greater than 2 weeks.

At study entry, all participants underwent rapid HIV testing with First Response® Rapid HIV 1–2-0 card tests (Premier Medical Corporation, Maharashtra, India). Discordant rapid tests were resolved with HIV ELISA via the GenScreen ULTRA HIV Ag-Ab Combo Kit and HIV was confirmed with the Geenius HIV-1/2 Supplemental Assay (Bio-Rad Laboratories, Hercules, CA). Hepatitis B surface antigen was detected using the GS HBsAg EIA 3.0 qualitative immunoassay (Bio-Rad Laboratories, Hercules, CA). Hepatitis C antibody testing was performed using the Ortho HCV version 3.0 ELISA Test System (Bio-Rad Laboratories, Hercules, CA). All testing was performed according to package inserts.

### Statistical analyses

Enrollment characteristics of the study population were summarized using descriptive statistics including medians with interquartile ranges (IQRs) for continuous variables and frequencies with percentages for categorical variables. Age was categorized as ≤22, 23–29, or ≥ 30 years based on preliminary data exploration to create relatively balanced groups and also align roughly with life-event stages. Because HIV testing is recommended at least yearly individuals at risk for HIV, our primary outcome of interest was a dichotomized variable for prior testing for HIV based on whether or not it had been conducted within the year prior to enrollment. PLWH who were aware of their HIV seropositivity prior to enrollment were excluded from analyses evaluating this outcome, since routine HIV testing would not be indicated in such participants, but were included in analyses of hepatitis and tuberculosis testing. Chi-squared or Fisher’s exact tests were used to evaluate differences between categorical groups of interest. We employed a robust Poisson model with a log link using a general linear modeling framework to estimate prevalence ratios (PRs) and 95% confidence intervals (CIs) for factors potentially associated with HIV testing in the year prior to enrollment. Participants with missing covariate data were excluded from analyses using listwise deletion as the total missingness constituted about 1% of participants. Variables that had a significance of *p* < 0.2 in unadjusted models were included in the multivariable model. This *p*-value threshold was used for down-selection of variables in order to liberally allow potential confounders into the multivariable model, reduce bias, and minimize type II error as compared to a more stringent selection criterion such as the *p* < 0.05 typically used to define statistical significance. Collinearity of variables included in the multivariable model was assessed via multiple means, including calculation of variance inflation factors for all variables modeled, calculation of the condition index values for the tolerance test, and examination of the Pearson correlation matrix. Lastly, in order to assess whether prior testing was associated with aspects of differential access due to spatial location, we constructed a 2-tier hierarchical model with level 1 as the individual respondent and level 2 as the residential ward. Residential wards were aggregated into 12 groups based on expert knowledge from in-country staff. Due to the number of geographic categories necessary to consider, the model was limited in the number of covariates that could be included as independent variables. Additionally, the comparison group for self-assessed HIV risk in the hierarchical model was modified to be the ‘some risk’ category as using the ‘no risk’ group resulted in model errors. All analyses were conducted in SAS 9.4; the hierarchical model was constructed using the GLMMIX environment.

## Results

### Study population and prevalence of testing uptake

From January 2017 to May 2018, 671 participants were enrolled in the study, including 52 (7.7%) PLWH and 619 (92.3%) without HIV. Their median age was 24 (IQR 21–28) years, 308 (45.9%) were female, and 508 (75.7%) were unmarried. The main occupations reported were sex worker (*n* = 150, 22.4%), fisherman (*n* = 119, 17.7%), business trader (*n* = 99, 14.7%), and construction (*n* = 41, 6.1%). PLWH tended to be older than participants without HIV (median 29 [IQR 27–30] vs 24 [IQR 21–28] years, *p* < 0.0001). There was a marginally higher proportion of females among PLWH as compared to participants without HIV (57.7% vs 44.9%, *p* = 0.08) but a smaller proportion of PLWH reported being married (42.3% vs 22.8%, *p* = 0.0016). The distribution of occupations did not differ significantly between participants with and without HIV (*p* = 0.36).

Any lifetime history of testing for HIV was reported by 651 (97.0%) participants, of whom 400 (61.2%) had been tested less than 6 months prior to enrollment, 129 (19.7%) had been tested 6–12 months prior to enrollment, and 125 (19.1%) had been tested more than 1 y prior to enrollment. Twenty-seven of these participants had a previous positive test result and were known to be living with HIV. An additional 24 participants were newly diagnosed with HIV upon enrollment into the cohort. Among participants without previously-diagnosed HIV, testing for HIV within the last year was more common among those who were female and had a secondary or higher level of education (Table 1). HIV testing was more common among participants in the “bar/pub/waitress” category than among other occupation categories.

**Table 1:** Characteristics of Study Participants, Stratified by History of Testing in the Year Prior to Enrollment

Characteristic	Tested for HIV^†^ (*n* = 644)		*p*	Tested for Hepatitis (*n* = 671)		***p***	Tested for Tuberculosis (*n* = 671)		***p***
	No	Yes		No	Yes		No	Yes	
	(*n* = 127)	(*n* = 517)		(*n* = 667)	(*n* = 4)		(*n* = 654)	(*n* = 17)	
Age									
≤ 22 years	48 (22)	174 (78)	0.29	225 (100)	0	0.14	219 (97)	6 (3)	1.00
23–29 years	52 (17)	251 (83)		313 (99)	4 (1)		309 (97)	8 (3)	
≥ 30 years	27 (23)	92 (77)		129 (100)	0		126 (98)	3 (2)	
Sex									
Male	81 (23)	275 (77)	**0.03**	360 (99)	3 (1)	0.63	354 (98)	9 (2)	1.00
Female	46 (16)	242 (84)		307 (100)	1(0)		300 (97)	8 (3)	
HIV Status^†^									
Living with HIV	–	–	**–**	52 (100)	0	1.00	47 (90)	5 (10)	
Without HIV	–	–		615 (99)	4 (1)		607 (98)	12 (2)	
Education Level									
Less than Secondary School	82 (23)	270 (77)	**0.01**	374 (100)	0	**0.04**	365 (98)	9 (2)	0.81
Secondary School or Higher	45 (15)	247 (85)		293 (99)	4 (1)		289 (97)	8 (3)	
Marital Status									
Single/Never Married	95 (19)	399 (81)	0.57	506 (100)	2 (0)	0.23	496 (98)	12 (2)	0.58
Married/Cohabitating	32 (21)	118 (79)		161 (99)	2 (1)		158 (97)	5 (3)	
Alcohol Abuse									
No	112 (19)	482 (81)	0.06	613 (99)	3 (1)	0.29	599 (97)	17 (3)	0.39
Yes	15 (30)	35 (70)		54 (98)	1 (2)		55 (100)	0	
Income									
≤ 9000 KSh	64 (20)	262 (80)	0.95	339 (99)	3 (1)	0.62	331 (97)	11 (23)	0.38
> 9000 KSh	63 (20)	255 (80)		328 (100)	1 (0)		323 (98)	6 (2)	
Occupation									
Sex Worker	24 (17)	121 (83)	**0.05**	149 (99)	1 (1)	1.00	147 (98)	3 (2)	0.64
Fisherman	32 (28)	82 (72)		118 (99)	1 (1)		117 (98)	2 (2)	
Bar/Pub/Waitress	8 (13)	53 (87)		64 (100)	0		61 (95)	3 (5)	
Other Employment	62 (19)	261 (81)		335 (99)	2 (1)		328 (97)	9 (3)	
Missing/Unknown*	1 (100)	0		1 (100)	0		1 (100)	0	
Self-Assessed HIV Risk									
No Risk	11 (19)	48 (81)	0.22	59 (100)	0	0.40	56 (95)	3 (5)	**0.002**
Some Risk	65 (18)	304 (82)		368 (100)	1 (0)		361 (98)	8 (2)	
High Risk	49 (24)	159 (76)		205 (99)	3 (1)		206 (99)	2 (1)	
Known to be living with HIV^†^	0	0		27 (100)	0		23 (85)	4 (15)	
Missing/Unknown*	2 (25)	6 (75)		8 (100)	0		8 (100)	0	

* Participants with “missing/unknown” data were excluded from analyses of the missing variable

†Twenty-seven participants were excluded from analyses of prior HIV testing because they were known to be living with HIV prior to enrollment in RV393 and repeated HIV testing is not indicated once the diagnosis has been established. For this reason, HIV status was not compared between groups with and without recent HIV testing

Prior testing was dichotomized based on whether or not it had been conducted within the year prior to enrollment. All data are presented as n (row %). Comparisons were made between groups using Pearson’s Chi-square test or, in cases with small cell sizes, Fisher’s exact test. Statistically significant *p*-values (*p* ≤ 0.05) are in **bold**

In contrast to HIV testing, prior testing for viral hepatitis and tuberculosis was uncommon. Any lifetime history of testing for viral hepatitis was reported by only 8 (1.2%) participants, of whom 1 (12.5%) had been tested less than 6 months prior to enrollment, 3 (37.5%) had been tested 6–12 months prior to enrollment, and 4 (50.0%) had been tested more than 1 y prior to enrollment. All participants with a history of testing for viral hepatitis were in the subgroup without HIV; none of the 52 PLWH enrolled in the study had ever been tested for viral hepatitis. At study entry, 28 (4.5%) participants without HIV had a positive hepatitis B surface antigen test and 21 (3.4%) had a reactive hepatitis C antibody, while 2 (3.9%) PLWH had a positive hepatitis B surface antigen test and none had a reactive hepatitis C antibody. No participants with a positive hepatitis test at study entry had any lifetime history of prior hepatitis testing.

For tuberculosis, 51 (7.6%) participants reported any lifetime history of testing, of whom 8 (15.7%) had been tested less than 6 months prior to enrollment, 9 (17.6%) had been tested 6–12 months prior to enrollment, and 34 (66.7%) had been tested more than 1 y prior to enrollment. A significantly higher proportion of PLWH participants reported any lifetime history of tuberculosis testing than was observed among participants without HIV (21.2% vs. 6.5%, Fisher *p* < 0.001). Participants with higher education were more likely to have been tested for viral hepatitis in the year prior to enrollment. There were no significant differences in characteristics between participants with and without a history of testing for tuberculosis in the year prior to enrollment (Table 1).

### Factors associated with HIV testing

Among participants without previously-diagnosed HIV, factors associated with prior testing for HIV within the year prior to enrollment were evaluated using robust Poisson regression models. Because prior testing for viral hepatitis and tuberculosis were each rare, factors associated with prior testing could not be evaluated using regression models. In unadjusted models, HIV testing in the past year was more common among females (PR 1.08 [95% CI 1.01, 1.17]) and participants with secondary education or higher (PR 1.10 [95% CI 1.02, 1.19]; Table 2). In the multivariable model, only secondary education or higher was associated with recent HIV testing (adjusted PR 1.10 [95% CI 1.02, 1.20]) after controlling for sex, alcohol use, and occupation. Occupations often associated with HIV risk in the region due to engagement in transactional sex were not associated with prior HIV testing in the multivariable model. No collinearity was observed among variables included in the multivariable model. Variance inflation factors for all variables modeled were below 1.2. Condition index values for the tolerance test were we well below the accepted cutoff of 30, with a maximum observed condition index of 7. Examination of the Pearson correlation matrix identified that no values were problematically correlated.

**Table 2:** Factors Associated with HIV Testing in the Year Prior to Enrollment of Adults at Risk for HIV in Kisumu, Kenya

Characteristic	Unadjusted Prevalence Ratio (95% Confidence Interval)	*p*	Adjusted Prevalence Ratio (95% Confidence Interval)	*p*
Age				
≤ 22 years	Reference		–	
23–29 years	1.06 (0.97, 1.15)	0.21	–	
≥ 30 years	0.99 (0.88, 1.11)	0.82	–	
Sex				
Male	Reference		Reference	
Female	**1.08 (1.01, 1.17)**	**0.03**	1.07 (0.96, 1.19)	0.23
Education Level				
Less than Secondary School	Reference		Reference	
Secondary School or Higher	**1.10 (1.02, 1.19)**	**0.01**	**1.10 (1.02, 1.20)**	**0.02**
Marital Status				
Single/Never Married	Reference		–	
Married/Cohabitating	0.97 (0.89, 1.07)	0.58	–	
Self-Assessed HIV Risk				
No Risk	Reference		–	
Some Risk	1.01 (0.89, 1.15)	0.85	–	
High Risk	0.94 (0.81, 1.08)	0.40	–	
Alcohol Abuse				
No	Reference		Reference	
Yes	0.86 (0.72, 1.04)	0.12	0.89 (0.74, 1.07)	0.21
Income				
≤ 9000	Reference		–	
> 9000	0.998 (0.92, 1.08)	0.95	–	
Occupation				
All Other Occupations	Reference		Reference	
Sex Worker	1.03 (0.94, 1.13)	0.48	1.01 (0.90, 1.13)	0.86
Fisherman	0.89 (0.78, 1.01)	0.07	0.96 (0.83, 1.10)	0.55
Bar/Pub/Waitress	1.08 (0.96, 1.20)	0.20	1.06 (0.94, 1.20)	0.34

Poisson regression with robust error variance was used to estimate prevalence ratios and 95% confidence intervals for factors potentially associated with prior testing for HIV in the year prior to study enrollment. Factors with *p* < 0.20 in unadjusted models were included in the adjusted model. Statistically significant (*p* < 0.05) prevalence ratios are shown in **bold**. Nine participants were excluded from the analysis through listwise deletion due to missing data for occupation and self-assessed HIV risk covariates

**Fig. 1:**
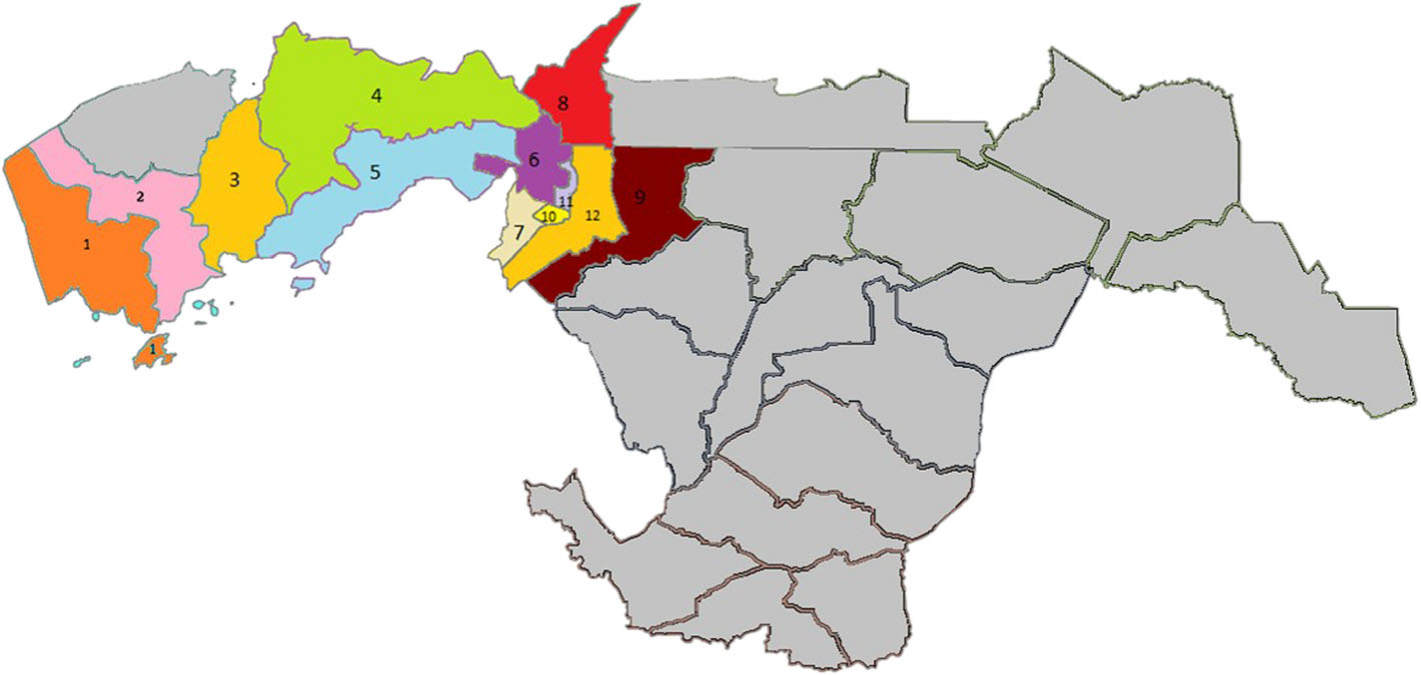
Aggregated participant residential locations in Kisumu County. Legend: 1 = West Seme; 2 = Central Seme; 3 = East Seme; 4 = Southwest, Central, and North Kisumu; 5 = West and Northwest Kisumu; 6 = Railways, Migosi, Shaurimoyo Kaloleni, Kondele; 7 = Market Milimani, Nayalenda B; 8 = Kjulu; 9 = Kolwa East; 10 = Manyatta B; 11 = Nyalenda A; 12 = Kolwa Central. Participant residential wards were aggregated into colored groups due to low N in some areas. Gray areas represent areas of Kisumu county wherein data collection for the RV393 study did not occur. This map was generated using ArcGIS v10.3.1 (www.arcgis.com, Environmental Systems Research Institute, Redlands, CA, USA) and edited in PowerPoint v16.0 and Paint v10.0 (www.microsoft.com, Microsoft Corporation, Redmond, WA, USA)

In the spatial location hierarchical models, Kisumu county was divided based on 12 separate sub-counties; participant location assignment was determined by self-reported household location (Fig. 1). Due to the number of distinct geographic groups, the model was limited in the number of independent variables that could be included in the model before convergence failure; for most combinations the model was limited to 2 independent variables. Numerous combinations of variables from the unadjusted and adjusted analysis were examined (data not shown), but there were no meaningful differences in the location effect on the models. None of the sub-county locations were significantly associated with prior testing uptake for HIV. As in the Poisson model, increased education level was associated with increased likelihood of recent HIV testing in the hierarchical model (PR 1.10 [95% CI 1.02, 1.20]; Table 3).

**Table 3:** Spatial Location Hierarchical Modelling of Factors Associated with HIV Testing in the Year Prior to Enrollment

Characteristic	Adjusted Prevalence Ratio (95% Confidence Interval)	*p*
*Fixed Effects*		
Education Level		
Less than Secondary School	Reference	
Secondary School or Higher	**1.10 (1.02, 1.20)**	**0.02**
Self-Assessed HIV Risk		
No Risk	0.98 (0.86, 1.11)	0.72
Some Risk	Reference	
High Risk	0.92 (0.84, 1.00)	0.06
*Random Effects*		
Area		
(1) West Seme	0.99 (0.91, 1.07)	0.78
(2) Central Seme	1.00 (0.96, 1.04)	0.92
(3) East Seme	1.00 (0.95, 1.04)	0.87
(4) Southwest, Central, and North Kisumu	1.00 (0.96, 1.04)	0.91
(5) West and Northwest Kisumu	1.00 (0.97, 1.04)	0.99
(6) Railways, Migosi, Shaurimoyo Kaloleni, Kondele	1.00 (0.97, 1.04)	0.91
(7) Market Milimani, Nayalenda B	1.01 (0.92, 1.10)	0.78
(8) Kajulu	0.99 (0.92, 1.07)	0.81
(9) Kolwa East	1.00 (0.96, 1.04)	0.95
(10) Manyatta B	1.00 (0.96, 1.03)	0.96
(11) Nyalenda A	1.01 (0.94, 1.07)	0.83
(12) Kolwa Central	1.00 (0.96, 1.04)	0.98
Overall Covariance Effect	1.00 (0.997, 1.004)	0.40

In order to assess whether prior testing was associated with aspects of differential access due to spatial location, we constructed a 2-tier hierarchical model with level 1 as the individual respondent and level 2 as the residential ward. Residential wards were aggregated into 12 groups based on expert knowledge from in-country staff as described in Fig. 1. Due to the number of geographic categories necessary to consider, the model was limited in the number of covariates that could be included as independent variables. Statistically significant (*p* < 0.05) prevalence ratios are shown in **bold**

## Discussion

The vast majority of participants in our cohort had been previously tested for HIV at some point in their lifetimes, as would be expected for a population selected based on known risk factors for HIV acquisition, but testing within the guideline-recommended annual timeframe was less common. However, there was a low prevalence of any prior testing for viral hepatitis and tuberculosis, either within the year prior to enrollment or more remotely.

By design, participants in our study had sexual behaviors that placed them at risk for HIV, HBV, and HCV. As such, they all should have been tested at least once in their lifetime for these diseases, with annual re-testing for HIV [17, 18]. Additionally, all PLWH should have been tested for HBV at entry into HIV care [18]. However, only 1.2% of participants in our study had ever been tested for viral hepatitis, including none of the participants living with HIV. These findings highlight substantial gaps in adherence to guideline-recommended screening practices that need to be addressed to optimize care, particularly for PLWH.

There was a trend toward increased likelihood of recent testing for HIV among females in our study as compared to males. These findings are similar to a 2017 assessment from Burkina Faso showing that males had lower testing uptake than females, but neither were meeting goals for testing [25]. Females benefit from additional touch points with healthcare systems, such as during antenatal care, that may facilitate HIV testing. In order to increase male participation in HIV testing, other interventions may be necessary. These can include peer-led interventions that have shown some efficacy in building trust with patients at risk for HIV [26]. Although females had higher testing uptake, they still are not meeting clinical goals for testing. Interventions should focus on both males and females in order to increase testing uptake in this population, though differentiated models of service delivery may be needed to reach each target population.

Higher education level was also associated with higher levels of prior testing for HIV. This is consistent with a number of recent studies from sub-Saharan Africa indicating that higher levels of education are positively associated with higher testing uptake [27, 28]. This is also consistent with prior studies in sub-Saharan Africa indicating that educational interventions aimed at high risk populations would potentially improve testing uptake.

Residential location appeared to have no bearing on prior testing within Kisumu county. This county borders Lake Victoria and is composed of rural and urban sub-counties. Our results showed no difference in testing between the 12 groupings of sub-counties evaluated. This appears to indicate that geographic or regional location is not an influential factor in testing uptake in Kisumu county, which further suggests that existing programs for testing have adequate—or at least consistent—coverage over the area. As location was not a significant influence on testing over the area, the effect estimates for covariates in the hierarchical model focused on geographic location did not differ meaningfully from the effects observed in the primary multivariable model.

Interestingly, there was no difference in HIV testing uptake in our cohort based on perceived risk of HIV. All participants in the study were considered to be at high risk for HIV based on their age and self-report of sex with multiple partners. Prior studies have demonstrated that people have a very difficult time self-identifying their own risks for HIV [29]. Health care providers must undertake objective risk stratification to better identify candidates for HIV testing.

Despite the benefits of routine screening and early treatment of HIV, viral hepatitis, and tuberculosis, there are significant barriers to testing uptake in Sub-Saharan Africa. These barriers include cost and resource constraints of the affected areas, perceived social stigma regarding either the testing or diagnosis [30], concern for loss of job, being treated poorly by health care workers, or potential intimate partner violence [21]. Our study found a number of populations that would benefit from targeted interventions to increase testing uptake. For HIV, these include men and persons with lower educational attainment. For viral hepatitis and tuberculosis, the low testing uptake across all demographics indicate this region would benefit from a regional approach to education and testing. Extensive HIV testing infrastructure could be leveraged to improve access to testing for other endemic infectious diseases.

This study described the uptake of testing for HIV, hepatitis, and tuberculosis during routine care prior to entry into a longitudinal study of participants with known risk factors for these diseases in Western Kenya. Despite a relatively large sample size, our analyses were not powered to detect small differences in testing patterns across geographic regions. Data were collected largely via self-report, which may be susceptible to biases including recall and social desirability bias. Questions about testing for viral hepatitis did not distinguish between HBV and HCV. Though tests for HIV, HBV, and HCV were conducted upon study entry, testing for tuberculosis was not systematically conducted as part of this study. Data on clinical screening for symptoms of tuberculosis were not collected in this study. The population of western Kenya may not be generalizable to other parts of sub-Saharan Africa.

## Conclusions

We found that testing for viral hepatitis and tuberculosis were uncommon in a population at risk for these diseases. While HIV testing within the last year was much more common, there were still some testing gaps. Females and persons with higher levels of education were more likely to have been tested for HIV recently, indicating that future interventions and research focusing on males and persons who have not finished secondary education may be productive. In our study, geography did not impact testing uptake indicating that HIV prevention services are uniform throughout the studied region. Self-assessed risk was poorly associated with HIV testing uptake, so providers need to be proactive and use objective criteria to identify candidates in need of HIV testing.

## Data Availability

The dataset supporting the conclusions of this article is available in the Harvard Dataverse repository, 10.7910/DVN/UANFUV .

## Abbreviations

CI: Confidence interval
HBV: Hepatitis B virus
HCV: Hepatitis C virus
HIV: Human immunodeficiency virus
IQR: Interquartile range
PLWH: People living with HIV
PR: Prevalence ratio
